# Supplementary material for: Overlapping cell population expression profiling and regulatory inference in C. elegans
Source: BMC Genomics. 2016 Feb 29;17:159. doi: 10.1186/s12864-016-2482-z (PMC4772325; doi:10.1186/s12864-016-2482-z)
Supplement: Additional file 13: — Web supplement. (DOC 21 kb) [file 12864_2016_2482_MOESM13_ESM.zip › sortWeb/clusters/hier.300.clusters/98.html]

Cluster 98 

## Cluster 98

### Expression

| cnd-1 rep. 1 | cnd-1 rep. 2 | cnd-1 rep. 3 | pha-4 rep. 1 | pha-4 rep. 2 | pha-4 rep. 3 | ceh-27 | ceh-36 | ceh-6 | F21D5.9 | mir-57 | mls-2 | pal-1 | pros-1 | ttx-3 | unc-130 | hlh-16 | irx-1 | ceh-6 (+) hlh-16 (+) | ceh-6 (+) hlh-16 (-) | ceh-6 (-) hlh-16 (+) | cnd-1 singlets | pha-4 singlets | 0 | 60 | 120 | 150 | 180 | 240 | 330 | 390 | 420 | 480 | 540 | 570 | 600 | 630 | 660 | NAME | Functional description |
| --- | --- | --- | --- | --- | --- | --- | --- | --- | --- | --- | --- | --- | --- | --- | --- | --- | --- | --- | --- | --- | --- | --- | --- | --- | --- | --- | --- | --- | --- | --- | --- | --- | --- | --- | --- | --- | --- | --- | --- |
|  |  |  |  |  |  |  |  |  |  |  |  |  |  |  |  |  |  |  |  |  |  |  |  |  |  |  |  |  |  |  |  |  |  |  |  |  |  | *let-653* | LEThal |
|  |  |  |  |  |  |  |  |  |  |  |  |  |  |  |  |  |  |  |  |  |  |  |  |  |  |  |  |  |  |  |  |  |  |  |  |  |  | *ptr-10* | PaTched Related family |
|  |  |  |  |  |  |  |  |  |  |  |  |  |  |  |  |  |  |  |  |  |  |  |  |  |  |  |  |  |  |  |  |  |  |  |  |  |  | *lpr-3* | LiPocalin-Related protein |
|  |  |  |  |  |  |  |  |  |  |  |  |  |  |  |  |  |  |  |  |  |  |  |  |  |  |  |  |  |  |  |  |  |  |  |  |  |  | *lpr-6* | LiPocalin-Related protein |
|  |  |  |  |  |  |  |  |  |  |  |  |  |  |  |  |  |  |  |  |  |  |  |  |  |  |  |  |  |  |  |  |  |  |  |  |  |  | *ptr-15* | PaTched Related family |
|  |  |  |  |  |  |  |  |  |  |  |  |  |  |  |  |  |  |  |  |  |  |  |  |  |  |  |  |  |  |  |  |  |  |  |  |  |  | F13D11.4 |  |
|  |  |  |  |  |  |  |  |  |  |  |  |  |  |  |  |  |  |  |  |  |  |  |  |  |  |  |  |  |  |  |  |  |  |  |  |  |  | C09F5.1 |  |
|  |  |  |  |  |  |  |  |  |  |  |  |  |  |  |  |  |  |  |  |  |  |  |  |  |  |  |  |  |  |  |  |  |  |  |  |  |  | *mboa-1* | Membrane Bound O-Acyl transferase, MBOAT |
|  |  |  |  |  |  |  |  |  |  |  |  |  |  |  |  |  |  |  |  |  |  |  |  |  |  |  |  |  |  |  |  |  |  |  |  |  |  | *mup-4* | MUscle Positioning |
|  |  |  |  |  |  |  |  |  |  |  |  |  |  |  |  |  |  |  |  |  |  |  |  |  |  |  |  |  |  |  |  |  |  |  |  |  |  | C11H1.9 |  |
|  |  |  |  |  |  |  |  |  |  |  |  |  |  |  |  |  |  |  |  |  |  |  |  |  |  |  |  |  |  |  |  |  |  |  |  |  |  | *clc-2* | CLaudin-like in Caenorhabditis |
|  |  |  |  |  |  |  |  |  |  |  |  |  |  |  |  |  |  |  |  |  |  |  |  |  |  |  |  |  |  |  |  |  |  |  |  |  |  | C37E2.2 |  |
|  |  |  |  |  |  |  |  |  |  |  |  |  |  |  |  |  |  |  |  |  |  |  |  |  |  |  |  |  |  |  |  |  |  |  |  |  |  | R09A8.5 |  |
|  |  |  |  |  |  |  |  |  |  |  |  |  |  |  |  |  |  |  |  |  |  |  |  |  |  |  |  |  |  |  |  |  |  |  |  |  |  | *atf-8* | ATF (cAMP-dependent transcription factor) family |
|  |  |  |  |  |  |  |  |  |  |  |  |  |  |  |  |  |  |  |  |  |  |  |  |  |  |  |  |  |  |  |  |  |  |  |  |  |  | *lpr-4* | LiPocalin-Related protein |
|  |  |  |  |  |  |  |  |  |  |  |  |  |  |  |  |  |  |  |  |  |  |  |  |  |  |  |  |  |  |  |  |  |  |  |  |  |  | C26B9.3 |  |
|  |  |  |  |  |  |  |  |  |  |  |  |  |  |  |  |  |  |  |  |  |  |  |  |  |  |  |  |  |  |  |  |  |  |  |  |  |  | *clc-5* | CLaudin-like in Caenorhabditis |
|  |  |  |  |  |  |  |  |  |  |  |  |  |  |  |  |  |  |  |  |  |  |  |  |  |  |  |  |  |  |  |  |  |  |  |  |  |  | ZK909.3 |  |
|  |  |  |  |  |  |  |  |  |  |  |  |  |  |  |  |  |  |  |  |  |  |  |  |  |  |  |  |  |  |  |  |  |  |  |  |  |  | *lpr-1* | LiPocalin-Related protein |
|  |  |  |  |  |  |  |  |  |  |  |  |  |  |  |  |  |  |  |  |  |  |  |  |  |  |  |  |  |  |  |  |  |  |  |  |  |  | C01B10.11 |  |
|  |  |  |  |  |  |  |  |  |  |  |  |  |  |  |  |  |  |  |  |  |  |  |  |  |  |  |  |  |  |  |  |  |  |  |  |  |  | *aqp-2* | AQuaPorin or aquaglyceroporin related |
|  |  |  |  |  |  |  |  |  |  |  |  |  |  |  |  |  |  |  |  |  |  |  |  |  |  |  |  |  |  |  |  |  |  |  |  |  |  | C24H10.1 |  |
|  |  |  |  |  |  |  |  |  |  |  |  |  |  |  |  |  |  |  |  |  |  |  |  |  |  |  |  |  |  |  |  |  |  |  |  |  |  | AH10.3 |  |
|  |  |  |  |  |  |  |  |  |  |  |  |  |  |  |  |  |  |  |  |  |  |  |  |  |  |  |  |  |  |  |  |  |  |  |  |  |  | T01D3.3 |  |
|  |  |  |  |  |  |  |  |  |  |  |  |  |  |  |  |  |  |  |  |  |  |  |  |  |  |  |  |  |  |  |  |  |  |  |  |  |  | F57G8.5 |  |
|  |  |  |  |  |  |  |  |  |  |  |  |  |  |  |  |  |  |  |  |  |  |  |  |  |  |  |  |  |  |  |  |  |  |  |  |  |  | *hst-3.1* | Heparan SulphoTransferase |
|  |  |  |  |  |  |  |  |  |  |  |  |  |  |  |  |  |  |  |  |  |  |  |  |  |  |  |  |  |  |  |  |  |  |  |  |  |  | ZC513.7 |  |
|  |  |  |  |  |  |  |  |  |  |  |  |  |  |  |  |  |  |  |  |  |  |  |  |  |  |  |  |  |  |  |  |  |  |  |  |  |  | R148.5 |  |
|  |  |  |  |  |  |  |  |  |  |  |  |  |  |  |  |  |  |  |  |  |  |  |  |  |  |  |  |  |  |  |  |  |  |  |  |  |  | *mlt-8* | MoLTing defective |
|  |  |  |  |  |  |  |  |  |  |  |  |  |  |  |  |  |  |  |  |  |  |  |  |  |  |  |  |  |  |  |  |  |  |  |  |  |  | B0454.6 |  |
|  |  |  |  |  |  |  |  |  |  |  |  |  |  |  |  |  |  |  |  |  |  |  |  |  |  |  |  |  |  |  |  |  |  |  |  |  |  | *perm-5* | PERMeable eggshell |
|  |  |  |  |  |  |  |  |  |  |  |  |  |  |  |  |  |  |  |  |  |  |  |  |  |  |  |  |  |  |  |  |  |  |  |  |  |  | F44E2.4 |  |
|  |  |  |  |  |  |  |  |  |  |  |  |  |  |  |  |  |  |  |  |  |  |  |  |  |  |  |  |  |  |  |  |  |  |  |  |  |  | *acn-1* | ACE (angiotensin converting enzyme)-like Non-peptidase |
|  |  |  |  |  |  |  |  |  |  |  |  |  |  |  |  |  |  |  |  |  |  |  |  |  |  |  |  |  |  |  |  |  |  |  |  |  |  | C34C12.6 |  |
|  |  |  |  |  |  |  |  |  |  |  |  |  |  |  |  |  |  |  |  |  |  |  |  |  |  |  |  |  |  |  |  |  |  |  |  |  |  | C52G5.2 |  |
|  |  |  |  |  |  |  |  |  |  |  |  |  |  |  |  |  |  |  |  |  |  |  |  |  |  |  |  |  |  |  |  |  |  |  |  |  |  | Y71G12B.25 |  |
|  |  |  |  |  |  |  |  |  |  |  |  |  |  |  |  |  |  |  |  |  |  |  |  |  |  |  |  |  |  |  |  |  |  |  |  |  |  | Y38H6C.16 |  |
|  |  |  |  |  |  |  |  |  |  |  |  |  |  |  |  |  |  |  |  |  |  |  |  |  |  |  |  |  |  |  |  |  |  |  |  |  |  | *lim-8* | LIM domain family |
|  |  |  |  |  |  |  |  |  |  |  |  |  |  |  |  |  |  |  |  |  |  |  |  |  |  |  |  |  |  |  |  |  |  |  |  |  |  | *soc-1* | Suppressor Of Clr |
|  |  |  |  |  |  |  |  |  |  |  |  |  |  |  |  |  |  |  |  |  |  |  |  |  |  |  |  |  |  |  |  |  |  |  |  |  |  | C44C1.1 |  |
|  |  |  |  |  |  |  |  |  |  |  |  |  |  |  |  |  |  |  |  |  |  |  |  |  |  |  |  |  |  |  |  |  |  |  |  |  |  | *dhs-27* | DeHydrogenases, Short chain |
|  |  |  |  |  |  |  |  |  |  |  |  |  |  |  |  |  |  |  |  |  |  |  |  |  |  |  |  |  |  |  |  |  |  |  |  |  |  | *let-4* | LEThal |
|  |  |  |  |  |  |  |  |  |  |  |  |  |  |  |  |  |  |  |  |  |  |  |  |  |  |  |  |  |  |  |  |  |  |  |  |  |  | *nhr-23* | Nuclear Hormone Receptor family |
|  |  |  |  |  |  |  |  |  |  |  |  |  |  |  |  |  |  |  |  |  |  |  |  |  |  |  |  |  |  |  |  |  |  |  |  |  |  | Y57G11C.31 |  |
|  |  |  |  |  |  |  |  |  |  |  |  |  |  |  |  |  |  |  |  |  |  |  |  |  |  |  |  |  |  |  |  |  |  |  |  |  |  | *nsy-4* | Neuronal SYmmetry |
|  |  |  |  |  |  |  |  |  |  |  |  |  |  |  |  |  |  |  |  |  |  |  |  |  |  |  |  |  |  |  |  |  |  |  |  |  |  | *inx-5* | INneXin |
|  |  |  |  |  |  |  |  |  |  |  |  |  |  |  |  |  |  |  |  |  |  |  |  |  |  |  |  |  |  |  |  |  |  |  |  |  |  | *inx-13* | INneXin |
|  |  |  |  |  |  |  |  |  |  |  |  |  |  |  |  |  |  |  |  |  |  |  |  |  |  |  |  |  |  |  |  |  |  |  |  |  |  | *inx-12* | INneXin |
|  |  |  |  |  |  |  |  |  |  |  |  |  |  |  |  |  |  |  |  |  |  |  |  |  |  |  |  |  |  |  |  |  |  |  |  |  |  | F56C11.5 |  |
|  |  |  |  |  |  |  |  |  |  |  |  |  |  |  |  |  |  |  |  |  |  |  |  |  |  |  |  |  |  |  |  |  |  |  |  |  |  | *hhat-1* | Homolog of Hedgehog AcylTransferase |
|  |  |  |  |  |  |  |  |  |  |  |  |  |  |  |  |  |  |  |  |  |  |  |  |  |  |  |  |  |  |  |  |  |  |  |  |  |  | *gly-6* | GLYcosylation related |
|  |  |  |  |  |  |  |  |  |  |  |  |  |  |  |  |  |  |  |  |  |  |  |  |  |  |  |  |  |  |  |  |  |  |  |  |  |  | *unc-23* | UNCoordinated |
|  |  |  |  |  |  |  |  |  |  |  |  |  |  |  |  |  |  |  |  |  |  |  |  |  |  |  |  |  |  |  |  |  |  |  |  |  |  | F23F1.7 |  |
|  |  |  |  |  |  |  |  |  |  |  |  |  |  |  |  |  |  |  |  |  |  |  |  |  |  |  |  |  |  |  |  |  |  |  |  |  |  | F31E3.8 |  |
|  |  |  |  |  |  |  |  |  |  |  |  |  |  |  |  |  |  |  |  |  |  |  |  |  |  |  |  |  |  |  |  |  |  |  |  |  |  | *ceh-1* | C. Elegans Homeobox |
|  |  |  |  |  |  |  |  |  |  |  |  |  |  |  |  |  |  |  |  |  |  |  |  |  |  |  |  |  |  |  |  |  |  |  |  |  |  | *mlt-7* | MoLTing defective |
|  |  |  |  |  |  |  |  |  |  |  |  |  |  |  |  |  |  |  |  |  |  |  |  |  |  |  |  |  |  |  |  |  |  |  |  |  |  | C09G9.3 |  |
|  |  |  |  |  |  |  |  |  |  |  |  |  |  |  |  |  |  |  |  |  |  |  |  |  |  |  |  |  |  |  |  |  |  |  |  |  |  | *hdl-2* | Histidine Decarboxyase Like |
|  |  |  |  |  |  |  |  |  |  |  |  |  |  |  |  |  |  |  |  |  |  |  |  |  |  |  |  |  |  |  |  |  |  |  |  |  |  | *nlp-12* | Neuropeptide-Like Protein |
|  |  |  |  |  |  |  |  |  |  |  |  |  |  |  |  |  |  |  |  |  |  |  |  |  |  |  |  |  |  |  |  |  |  |  |  |  |  | Y57G11B.1 |  |
|  |  |  |  |  |  |  |  |  |  |  |  |  |  |  |  |  |  |  |  |  |  |  |  |  |  |  |  |  |  |  |  |  |  |  |  |  |  | *hsp-12.3* | Heat Shock Protein |
|  |  |  |  |  |  |  |  |  |  |  |  |  |  |  |  |  |  |  |  |  |  |  |  |  |  |  |  |  |  |  |  |  |  |  |  |  |  | *anmt-2* | Amine N-MethylTransferase |
|  |  |  |  |  |  |  |  |  |  |  |  |  |  |  |  |  |  |  |  |  |  |  |  |  |  |  |  |  |  |  |  |  |  |  |  |  |  | *hsp-25* | Heat Shock Protein |
|  |  |  |  |  |  |  |  |  |  |  |  |  |  |  |  |  |  |  |  |  |  |  |  |  |  |  |  |  |  |  |  |  |  |  |  |  |  | Y57G7A.11 |  |
|  |  |  |  |  |  |  |  |  |  |  |  |  |  |  |  |  |  |  |  |  |  |  |  |  |  |  |  |  |  |  |  |  |  |  |  |  |  | Y82E9BR.20 |  |
|  |  |  |  |  |  |  |  |  |  |  |  |  |  |  |  |  |  |  |  |  |  |  |  |  |  |  |  |  |  |  |  |  |  |  |  |  |  | *ras-2* | R-RAS related |
|  |  |  |  |  |  |  |  |  |  |  |  |  |  |  |  |  |  |  |  |  |  |  |  |  |  |  |  |  |  |  |  |  |  |  |  |  |  | C44B12.3 |  |
|  |  |  |  |  |  |  |  |  |  |  |  |  |  |  |  |  |  |  |  |  |  |  |  |  |  |  |  |  |  |  |  |  |  |  |  |  |  | *dhs-29* | DeHydrogenases, Short chain |
|  |  |  |  |  |  |  |  |  |  |  |  |  |  |  |  |  |  |  |  |  |  |  |  |  |  |  |  |  |  |  |  |  |  |  |  |  |  | M60.4 |  |
|  |  |  |  |  |  |  |  |  |  |  |  |  |  |  |  |  |  |  |  |  |  |  |  |  |  |  |  |  |  |  |  |  |  |  |  |  |  | *dpy-10* | DumPY: shorter than wild-type |
|  |  |  |  |  |  |  |  |  |  |  |  |  |  |  |  |  |  |  |  |  |  |  |  |  |  |  |  |  |  |  |  |  |  |  |  |  |  | C24H10.3 |  |
|  |  |  |  |  |  |  |  |  |  |  |  |  |  |  |  |  |  |  |  |  |  |  |  |  |  |  |  |  |  |  |  |  |  |  |  |  |  | *tyr-2* | TYRosinase |
|  |  |  |  |  |  |  |  |  |  |  |  |  |  |  |  |  |  |  |  |  |  |  |  |  |  |  |  |  |  |  |  |  |  |  |  |  |  | K09E3.7 |  |
|  |  |  |  |  |  |  |  |  |  |  |  |  |  |  |  |  |  |  |  |  |  |  |  |  |  |  |  |  |  |  |  |  |  |  |  |  |  | H14A12.8 |  |
|  |  |  |  |  |  |  |  |  |  |  |  |  |  |  |  |  |  |  |  |  |  |  |  |  |  |  |  |  |  |  |  |  |  |  |  |  |  | F10D7.3 |  |
|  |  |  |  |  |  |  |  |  |  |  |  |  |  |  |  |  |  |  |  |  |  |  |  |  |  |  |  |  |  |  |  |  |  |  |  |  |  | *str-138* | Seven TM Receptor |
|  |  |  |  |  |  |  |  |  |  |  |  |  |  |  |  |  |  |  |  |  |  |  |  |  |  |  |  |  |  |  |  |  |  |  |  |  |  | F54C9.14 |  |
|  |  |  |  |  |  |  |  |  |  |  |  |  |  |  |  |  |  |  |  |  |  |  |  |  |  |  |  |  |  |  |  |  |  |  |  |  |  | C05E11.6 |  |
|  |  |  |  |  |  |  |  |  |  |  |  |  |  |  |  |  |  |  |  |  |  |  |  |  |  |  |  |  |  |  |  |  |  |  |  |  |  | *clec-166* | C-type LECtin |
|  |  |  |  |  |  |  |  |  |  |  |  |  |  |  |  |  |  |  |  |  |  |  |  |  |  |  |  |  |  |  |  |  |  |  |  |  |  | *lact-7* | beta-LACTamase domain containing |
|  |  |  |  |  |  |  |  |  |  |  |  |  |  |  |  |  |  |  |  |  |  |  |  |  |  |  |  |  |  |  |  |  |  |  |  |  |  | K08E3.2 |  |
|  |  |  |  |  |  |  |  |  |  |  |  |  |  |  |  |  |  |  |  |  |  |  |  |  |  |  |  |  |  |  |  |  |  |  |  |  |  | Y5H2B.8 |  |
|  |  |  |  |  |  |  |  |  |  |  |  |  |  |  |  |  |  |  |  |  |  |  |  |  |  |  |  |  |  |  |  |  |  |  |  |  |  | *col-184* | COLlagen |
|  |  |  |  |  |  |  |  |  |  |  |  |  |  |  |  |  |  |  |  |  |  |  |  |  |  |  |  |  |  |  |  |  |  |  |  |  |  | ZC64.7 |  |
|  |  |  |  |  |  |  |  |  |  |  |  |  |  |  |  |  |  |  |  |  |  |  |  |  |  |  |  |  |  |  |  |  |  |  |  |  |  | Y39A3CL.13 |  |
|  |  |  |  |  |  |  |  |  |  |  |  |  |  |  |  |  |  |  |  |  |  |  |  |  |  |  |  |  |  |  |  |  |  |  |  |  |  | Y57G11C.t1 |  |
|  |  |  |  |  |  |  |  |  |  |  |  |  |  |  |  |  |  |  |  |  |  |  |  |  |  |  |  |  |  |  |  |  |  |  |  |  |  | *wht-4* | WHiTe (Drosophila) related ABC transporter |
|  |  |  |  |  |  |  |  |  |  |  |  |  |  |  |  |  |  |  |  |  |  |  |  |  |  |  |  |  |  |  |  |  |  |  |  |  |  | *ugt-15* | UDP-GlucuronosylTransferase |
|  |  |  |  |  |  |  |  |  |  |  |  |  |  |  |  |  |  |  |  |  |  |  |  |  |  |  |  |  |  |  |  |  |  |  |  |  |  | C08E3.1 |  |
|  |  |  |  |  |  |  |  |  |  |  |  |  |  |  |  |  |  |  |  |  |  |  |  |  |  |  |  |  |  |  |  |  |  |  |  |  |  | Y82E9BR.10 |  |
|  |  |  |  |  |  |  |  |  |  |  |  |  |  |  |  |  |  |  |  |  |  |  |  |  |  |  |  |  |  |  |  |  |  |  |  |  |  | *grd-10* | GRounDhog (hedgehog-like family) |
|  |  |  |  |  |  |  |  |  |  |  |  |  |  |  |  |  |  |  |  |  |  |  |  |  |  |  |  |  |  |  |  |  |  |  |  |  |  | C54E10.t2 |  |
|  |  |  |  |  |  |  |  |  |  |  |  |  |  |  |  |  |  |  |  |  |  |  |  |  |  |  |  |  |  |  |  |  |  |  |  |  |  | M01G5.9 |  |
|  |  |  |  |  |  |  |  |  |  |  |  |  |  |  |  |  |  |  |  |  |  |  |  |  |  |  |  |  |  |  |  |  |  |  |  |  |  | F11A6.7 |  |
|  |  |  |  |  |  |  |  |  |  |  |  |  |  |  |  |  |  |  |  |  |  |  |  |  |  |  |  |  |  |  |  |  |  |  |  |  |  | *rte-2* | tRNA (RNA, Transfer), glutamic acid (E) |
|  |  |  |  |  |  |  |  |  |  |  |  |  |  |  |  |  |  |  |  |  |  |  |  |  |  |  |  |  |  |  |  |  |  |  |  |  |  | *lips-10* | LIPaSe related |
|  |  |  |  |  |  |  |  |  |  |  |  |  |  |  |  |  |  |  |  |  |  |  |  |  |  |  |  |  |  |  |  |  |  |  |  |  |  | F48G7.4 |  |
|  |  |  |  |  |  |  |  |  |  |  |  |  |  |  |  |  |  |  |  |  |  |  |  |  |  |  |  |  |  |  |  |  |  |  |  |  |  | Y51A2D.32 |  |
|  |  |  |  |  |  |  |  |  |  |  |  |  |  |  |  |  |  |  |  |  |  |  |  |  |  |  |  |  |  |  |  |  |  |  |  |  |  | Y71A12B.15 |  |
|  |  |  |  |  |  |  |  |  |  |  |  |  |  |  |  |  |  |  |  |  |  |  |  |  |  |  |  |  |  |  |  |  |  |  |  |  |  | Y38H6C.15 |  |
|  |  |  |  |  |  |  |  |  |  |  |  |  |  |  |  |  |  |  |  |  |  |  |  |  |  |  |  |  |  |  |  |  |  |  |  |  |  | Y105C5A.14 |  |
|  |  |  |  |  |  |  |  |  |  |  |  |  |  |  |  |  |  |  |  |  |  |  |  |  |  |  |  |  |  |  |  |  |  |  |  |  |  | F21G4.1 |  |
|  |  |  |  |  |  |  |  |  |  |  |  |  |  |  |  |  |  |  |  |  |  |  |  |  |  |  |  |  |  |  |  |  |  |  |  |  |  | K06A1.2 |  |
|  |  |  |  |  |  |  |  |  |  |  |  |  |  |  |  |  |  |  |  |  |  |  |  |  |  |  |  |  |  |  |  |  |  |  |  |  |  | *cutl-16* | CUTiclin-Like |
|  |  |  |  |  |  |  |  |  |  |  |  |  |  |  |  |  |  |  |  |  |  |  |  |  |  |  |  |  |  |  |  |  |  |  |  |  |  | *cutl-9* | CUTiclin-Like |
|  |  |  |  |  |  |  |  |  |  |  |  |  |  |  |  |  |  |  |  |  |  |  |  |  |  |  |  |  |  |  |  |  |  |  |  |  |  | C06E7.4 |  |
|  |  |  |  |  |  |  |  |  |  |  |  |  |  |  |  |  |  |  |  |  |  |  |  |  |  |  |  |  |  |  |  |  |  |  |  |  |  | *nhr-25* | Nuclear Hormone Receptor family |
|  |  |  |  |  |  |  |  |  |  |  |  |  |  |  |  |  |  |  |  |  |  |  |  |  |  |  |  |  |  |  |  |  |  |  |  |  |  | *fkb-7* | FK506-Binding protein family |
|  |  |  |  |  |  |  |  |  |  |  |  |  |  |  |  |  |  |  |  |  |  |  |  |  |  |  |  |  |  |  |  |  |  |  |  |  |  | *mua-3* | MUscle Attachment abnormal |
|  |  |  |  |  |  |  |  |  |  |  |  |  |  |  |  |  |  |  |  |  |  |  |  |  |  |  |  |  |  |  |  |  |  |  |  |  |  | F53F1.2 |  |
|  |  |  |  |  |  |  |  |  |  |  |  |  |  |  |  |  |  |  |  |  |  |  |  |  |  |  |  |  |  |  |  |  |  |  |  |  |  | F32A5.8 |  |
|  |  |  |  |  |  |  |  |  |  |  |  |  |  |  |  |  |  |  |  |  |  |  |  |  |  |  |  |  |  |  |  |  |  |  |  |  |  | F32A5.9 |  |
|  |  |  |  |  |  |  |  |  |  |  |  |  |  |  |  |  |  |  |  |  |  |  |  |  |  |  |  |  |  |  |  |  |  |  |  |  |  | F53B3.5 |  |
|  |  |  |  |  |  |  |  |  |  |  |  |  |  |  |  |  |  |  |  |  |  |  |  |  |  |  |  |  |  |  |  |  |  |  |  |  |  | E01G4.5 |  |
|  |  |  |  |  |  |  |  |  |  |  |  |  |  |  |  |  |  |  |  |  |  |  |  |  |  |  |  |  |  |  |  |  |  |  |  |  |  | Y40A1A.3 |  |

### Phenotypes enriched

none found

### Anatomy terms enriched

|  |  |  |  |
| --- | --- | --- | --- |
| **Group name** | **Number in cluster** | **Enrichment** | **FDR corrected p** |
| OLLsoR | 4 | 30.31 | 0.0155 |
| OLLsoL | 4 | 30.31 | 0.0155 |
| seam cell | 13 | 4.10 | 0.0302 |
| hypodermal cell | 15 | 3.59 | 0.0311 |
| epithelial cell | 18 | 3.00 | 0.0430 |

### GO terms enriched

|  |  |  |
| --- | --- | --- |
| **GO term** | **Number of genes** | **FDR-corrected p-value** |
| molting cycle, collagen and cuticulin-based cuticle | 9 | 0.012 |
| lipid binding | 6 | 0.048 |

### Expression clusters enriched

|  |  |  |  |
| --- | --- | --- | --- |
| **Group name** | **Number in cluster** | **Enrichment** | **FDR corrected p** |
| Genes that show selective expression in a subset of cell types vs broadly expressed in many cell types. Correspond to 20% - 57% of enriched\_genes for a given cell type. WBPaper00037950:hypodermis\_embryo\_SelectivelyEnriched | 26 | 9.90 | 8.24e-16 |
| Genes dowm regulated in the dauer versus dauer-exit worms. | 26 | 8.10 | 1.01e-13 |
| Genes that showed lower expression in N2 than in DR1350. | 38 | 4.42 | 1.38e-12 |
| Genes down regulated in the absence of TDP-1, when the threshold was set at a fold change (FC) of 1.5. | 22 | 7.01 | 4.33e-10 |
| Germline-enriched and sex-biased expression profile cluster B. | 28 | 5.08 | 5.25e-10 |
| Genes significantly enriched (> 2x, FDR < 5%) in a particular cell-type versus a reference sample of all cells at the same stage. WBPaper00037950:hypodermis\_embryo\_enriched | 27 | 5.02 | 1.85e-09 |
| Genes with expression level up regulated after treatment with Methylmercury (MeHg) by RNAseq analysis. | 23 | 5.97 | 3.00e-09 |
| Genes that showed decreased expression in nhr-23 RNAi experiment. | 17 | 8.85 | 5.58e-09 |
| Genes that show selective expression in a subset of cell types vs broadly expressed in many cell types. Correspond to 20% - 57% of enriched\_genes for a given cell type. WBPaper00037950:hypodermis\_larva\_SelectivelyEnriched | 23 | 5.32 | 2.89e-08 |
| Genes upregulated in dcr-1(-/-) adult animals by at least 1.5 fold and P < 0.01, as determined by a multisample t-test and the Benjamini and Hochberg false discovery rate correction. | 30 | 3.80 | 7.85e-08 |
| Genes up regulated by mir-243(n4759). | 39 | 2.95 | 1.16e-07 |
| Genes up regulated in crh-1(nn3315) comparing to in N2. | 20 | 4.91 | 2.04e-06 |
| Genes down regulated in the absence of TDP-1, when the threshold was set at a fold change (FC) of 1.2. | 43 | 2.46 | 2.55e-06 |
| WT-Pico Pan-neural Depleted Genes, with genes found multiple times in a single dataset removed (without dups). | 30 | 3.25 | 2.60e-06 |
| Genes down-regulated after 100 um Quercetin treatment. Fold change < 0.8. | 29 | 3.26 | 4.70e-06 |
| Genes depleted in muscle cells (0hr muscle dataset). Dissociated myo-3::GFP embryos were cultured for 0 hours before FACS sorting. | 34 | 2.71 | 1.60e-05 |
| Genes upregulated in sma-2 L4 (3 arrays) or sma-4 L4 (1 array) vs. N2 L4. | 18 | 4.74 | 1.93e-05 |
| Genes down-regulated after 200 um Quercetin treatment. Fold change < 0.8. | 29 | 3.03 | 2.06e-05 |
| C-lineage related expression profile. WBPaper00025032:cluster\_24 | 7 | 20.31 | 2.24e-05 |
| Genes significantly enriched (> 2x, FDR < 5%) in a particular cell-type versus a reference sample of all cells at both embryonic and larval stages. WBPaper00037950:hypodermis\_CoreEnriched | 11 | 8.48 | 3.32e-05 |
| Genes depleted in muscle cells (24hr muscle dataset). Dissociated myo-3::GFP embryos were cultured for 24 hours before FACS sorting. | 32 | 2.75 | 3.38e-05 |
| Total muscle depleted genes (complete list of non-overlapping genes from the 0hr and 24hr muscle depleted datasets). | 41 | 2.25 | 7.06e-05 |
| A cluster of genes that oscillates with the molting cycle during early larval development. | 11 | 7.32 | 1.33e-04 |
| Genes that showed lower expression in RIL-17 than in RIL-14. | 9 | 9.82 | 1.41e-04 |
| Genes regulated by octr-1(ok371) after infected with P. aeruginosa PA14 for 4 hours at 25 centigrade. | 23 | 3.21 | 2.13e-04 |
| Genes significantly enriched (> 2x, FDR < 5%) in a particular cell-type versus a reference sample of all cells at the same stage. WBPaper00037950:hypodermis\_larva\_enriched | 26 | 2.83 | 3.62e-04 |
| Gene significantly up-regulated by treatment with 0.2mM of HuminFeed until older adult stage (11 days), with a minimum fold change in gene expression of 1.25. | 14 | 4.53 | 8.79e-04 |
| Developmentally modulated gene cluster. cgc4386\_cluster\_5\_6 | 7 | 11.36 | 9.59e-04 |
| Genes downregulated on Comamonas DA1877 relative to E. coli OP50, Young adult | 12 | 5.20 | 1.19e-03 |
| Genes downregulated by fer-1 mutants hc1 and hc24, with > 4 fold change in expression level. | 10 | 6.23 | 1.60e-03 |
| Genes regulated by heat shock. | 18 | 3.32 | 2.25e-03 |
| Genes with variation in expression level across genotypes (AB2, CB4856, RC301, CB4857, N2, HC445). | 32 | 2.21 | 2.77e-03 |
| Expression Pattern Group C, enriched for genes involved in metabolic processes. | 21 | 2.89 | 2.94e-03 |
| A large cluster of genes up-regulated during early larval development.. | 30 | 2.23 | 4.68e-03 |
| Genome-wide analysis of developmental and sex-regulated gene expression profile. cgc4489\_group\_8 | 15 | 3.61 | 4.90e-03 |
| Genes with increased expression after 24 hours of infection by P.lumniescens Fold changes shown are pathogen vs OP50. WBPaper00038438:P.lumniescens\_24hr\_upregulated\_TilingArray | 48 | 1.73 | 6.17e-03 |
| Up-regulated genes under 0.5mg/l CPF treatment at 16 centigrade. | 8 | 6.78 | 6.83e-03 |
| miRNA targets that are significantly enriched at embryo stage. To generate a global view of the dynamics of miRNA-mediated regulation of gene expression during C. elegans development, authors analyzed the mRNAs in the AIN-2-GFP IP results from five developmental stages. The magnitude of the combined interaction of miRNAs with a given target mRNA was assessed by measuring the fold enrichment of that mRNA in AIN-2 IP samples, relative to the abundance of the mRNA in the corresponding total lysate. Because this enrichment in the IP sample versus total lysate directly reflects the miRISC-associated fraction of a given mRNA, high enrichment indicates the likelihood of strong miRNA-mediated regulation of the mRNA, whereas low or negative enrichment indicates the likelihood of weak or absent miRNA regulation of the mRNA. It is also possible that poor enrichment could reflect interactions that occur only in a rare subset of cells at any given stage of development. Transcripts that were significantly enriched (0 | 17 | 3.15 | 7.20e-03 |
| Genes down regulated in aak-2 over expression line uthIs202 comparing to in N2. | 11 | 4.43 | 1.05e-02 |
| Class E gene expression showed no change in lin-14(lf) in L1, up regulation in lin-4(lf) in L2. | 10 | 4.06 | 4.05e-02 |

### Motifs enriched

|  |  |  |  |  |  |
| --- | --- | --- | --- | --- | --- |
| **Motif** | **Logo** | **Possible orthologs** | **Number of motifs in cluster** | **Enrichment** | **FDR corrected p** |
| pTH3811 |  | nhr-19 (0.59) nhr-2 (-0.58) nhr-69 (0.55) nhr-10 nhr-213 | 62 | 2.88 | 1.4e-13 |
| pTH6589 |  | nhr-2 (-0.58) nhr-15 nhr-239 nhr-213 | 41 | 3.88 | 1.7e-11 |
| MA0071.1 |  | nhr-2 (-0.58) nhr-10 nhr-6 nhr-68 nhr-213 nhr-71 Y67D8A.3 | 29 | 5.74 | 2.7e-11 |
| pTH4269 |  | nhr-2 (-0.58) F13H6.1 nhr-86 nhr-177 | 40 | 3.77 | 8.4e-11 |
| RORA\_f1 |  | nhr-213 nhr-118 | 40 | 3.76 | 9.4e-11 |
| STF1\_f1 |  | nhr-68 | 38 | 3.87 | 1.9e-10 |
| MA0599.1 |  | klf-1 klf-2 ZC328.2 | 87 | 1.74 | 2.6e-09 |
| NR2F1\_4 |  | nhr-19 (0.59) nhr-2 (-0.58) nhr-62 | 65 | 2.05 | 5.8e-08 |
| SP4\_1 |  | klf-1 klf-2 | 53 | 2.26 | 3.7e-07 |
| pTH10722 |  | eor-1 (-0.52) ref-2 egrh-3 | 68 | 1.85 | 1.1e-06 |
| MA0543.1 |  | eor-1 (-0.52) daf-8 | 57 | 2.05 | 1.9e-06 |
| Hnf4a\_2640 |  | nhr-62 | 46 | 2.35 | 2.4e-06 |
| pTH3086 |  | klf-1 sptf-3 klf-2 | 67 | 1.81 | 3.8e-06 |
| pTH5714 |  | nhr-239 | 65 | 1.84 | 4.5e-06 |
| V$CREB\_01 |  | crh-1 attf-1 | 51 | 2.15 | 4.5e-06 |
| GM12878\_PBX3\_HudsonAlpha |  | nfya-2 (-0.63) lin-31 cey-3 dro-1 ceh-20 | 49 | 2.16 | 8.1e-06 |
| K562\_ZBTB7A\_HudsonAlpha |  | ZC328.2 | 89 | 1.47 | 1.3e-05 |
| pTH6071 |  | C46E10.8 C33G8.2 | 60 | 1.87 | 1.5e-05 |
| I$KR\_01 |  | B0310.2 (0.6) ZK177.3 | 78 | 1.59 | 1.6e-05 |
| ECC-1\_ERALPHA\_HudsonAlpha |  | nhr-43 (0.59) nhr-6 nhr-71 | 60 | 1.85 | 1.9e-05 |
| pTH5561 |  | nhr-239 | 65 | 1.77 | 2.0e-05 |
| MA0508.1 |  | blmp-1 (0.91) | 50 | 2.05 | 2.4e-05 |
| ELF2\_f1 |  | lin-1 C24A1.2 | 110 | 1.23 | 2.8e-05 |
| pTH5437 |  | ceh-34 | 43 | 2.24 | 2.9e-05 |
| PURA\_f1 |  | Y53H1A.2 klf-2 plp-2 | 46 | 2.14 | 3.0e-05 |
| srp\_FlyReg\_FBgn0003507 |  | lin-39 elt-1 | 57 | 1.88 | 3.3e-05 |
| V$GATA1\_03 |  | elt-1 | 43 | 2.23 | 3.4e-05 |
| EVX2\_1 |  | dsc-1 (0.69) egl-5 (0.61) ceh-1 (0.56) lim-4 (-0.56) cog-1 (0.53) ceh-36 (-0.52) ceh-43 ceh-14 lim-6 pal-1 lim-7 lin-39 ceh-53 ceh-45 ceh-16 ceh-23 ceh-10 ceh-30 mls-2 alr-1 and 7 others  [full list] | 55 | 1.91 | 3.7e-05 |
| K562\_EGR1\_HudsonAlpha |  | klf-1 klf-2 ZC328.2 | 67 | 1.68 | 7.0e-05 |
| Sox30\_2781 |  | gei-3 sox-4 pop-1 C05C9.3 | 57 | 1.83 | 7.1e-05 |
| Foxg1\_2 |  | lin-31 nhr-213 | 57 | 1.83 | 7.7e-05 |
| pTH8991 |  | cey-3 | 35 | 2.45 | 8.3e-05 |
| pTH1001 |  | dnj-17 | 97 | 1.34 | 9.4e-05 |
| EHF\_si |  | nhr-19 (0.59) lin-1 C24A1.2 | 79 | 1.51 | 1.1e-04 |
| FLI1\_2 |  | lin-1 | 46 | 2.04 | 1.1e-04 |
| MA0163.1 |  | Y53H1A.2 D1081.8 | 88 | 1.42 | 1.1e-04 |
| V$FAC1\_01 |  | gei-8 | 75 | 1.55 | 1.2e-04 |
| Mcm1 |  | unc-120 (0.55) | 56 | 1.81 | 1.4e-04 |
| MA0459.1 |  | nhr-2 (-0.58) nhr-239 nhr-62 | 76 | 1.54 | 1.4e-04 |
| pTH3477 |  | daf-16 | 44 | 2.06 | 1.7e-04 |
| pTH6445 |  | ceh-5 | 54 | 1.83 | 1.8e-04 |
| FOXO6\_3 |  | daf-16 ZC328.2 | 104 | 1.26 | 1.9e-04 |
| pTH9222 |  | mel-28 | 64 | 1.67 | 1.9e-04 |
| SMAD3\_f1 |  | daf-8 | 59 | 1.74 | 2.0e-04 |
| I$FTZ\_01 |  | ceh-1 (0.56) cog-1 (0.53) ceh-43 lim-7 lin-39 ceh-9 ceh-8 ceh-30 ceh-24 alr-1 ceh-31 ceh-19 | 55 | 1.80 | 2.3e-04 |
| V$TAXCREB\_02 |  | crh-1 zip-3 | 51 | 1.87 | 2.3e-04 |
| Ehf |  | lin-1 C24A1.2 | 56 | 1.78 | 2.3e-04 |
| MA0165.1 |  | php-3 ceh-24 ceh-13 D1005.3 | 71 | 1.57 | 2.5e-04 |
| V$HOX13\_01 |  | lin-39 | 67 | 1.62 | 2.5e-04 |
| PAX6\_1 |  | pax-3 pax-2 | 42 | 2.08 | 2.6e-04 |
| pTH5423 |  | klf-2 | 65 | 1.64 | 2.6e-04 |
| Sox15\_3457 |  | sox-4 | 53 | 1.82 | 2.7e-04 |
| Hoxd8\_2644 |  | lin-39 | 56 | 1.76 | 3.1e-04 |
| pTH10714 |  | nhr-216 nhr-84 nhr-142 | 64 | 1.64 | 3.1e-04 |
| Gm4881 |  | lin-1 F19F10.1 C24A1.2 | 50 | 1.86 | 3.4e-04 |
| pTH10823 |  | B0310.2 (0.6) | 102 | 1.27 | 3.4e-04 |
| pTH9080 |  | mnm-2 | 58 | 1.72 | 3.5e-04 |
| MA0470.1 |  | efl-1 F49E12.6 | 101 | 1.28 | 3.7e-04 |
| EN1\_1 |  | ceh-1 (0.56) ceh-43 lim-7 ceh-9 lin-39 ceh-16 alr-1 ceh-31 | 44 | 1.99 | 3.8e-04 |
| ETS2\_f1 |  | lin-1 C24A1.2 | 83 | 1.43 | 3.8e-04 |
| Elf3\_3876 |  | C24A1.2 | 57 | 1.73 | 3.9e-04 |
| I$MTTFA\_01 |  | hmg-5 | 105 | 1.24 | 3.9e-04 |
| ZNF75A\_1 |  | ztf-3 F26F4.8 lag-1 | 95 | 1.33 | 4.0e-04 |
| pTH2283 |  | odd-2 | 69 | 1.57 | 4.1e-04 |
| Dlx5\_3419 |  | ceh-1 (0.56) npax-3 ceh-43 lin-39 ceh-53 ceh-45 alr-1 ceh-18 ceh-12 | 45 | 1.95 | 4.2e-04 |
| pTH5887 |  | lin-39 | 95 | 1.32 | 4.3e-04 |
| N$DAF16\_01 |  | lin-31 let-381 daf-16 fkh-7 fkh-10 fkh-8 | 71 | 1.55 | 4.3e-04 |
| pTH3998 |  | tbx-39 | 58 | 1.71 | 4.3e-04 |
| HepG2\_HSF1\_Stanford |  | Y53C10A.3 | 22 | 3.08 | 4.6e-04 |
| bin\_SANGER\_5\_FBgn0045759 |  | lin-31 let-381 daf-16 fkh-7 fkh-8 fkh-10 pha-4 | 49 | 1.86 | 4.8e-04 |
| pTH6423 |  | pha-2 | 68 | 1.57 | 4.8e-04 |
| ZBTB7A\_1 |  | ref-2 pax-3 ztf-14 ZC328.2 | 40 | 2.07 | 4.9e-04 |
| V$TCF11\_01 |  | skn-1 | 59 | 1.68 | 5.2e-04 |
| Hmx3\_3490 |  | ceh-9 | 46 | 1.91 | 5.3e-04 |
| Egr1\_2580 |  | ZC328.2 | 63 | 1.63 | 5.4e-04 |
| PDX1\_do |  | ceh-43 pal-1 lin-39 alr-1 ceh-18 ceh-12 | 51 | 1.81 | 5.4e-04 |
| MA0476.1 |  | fos-1 jun-1 crh-1 | 32 | 2.35 | 5.5e-04 |
| RFX1\_f1 |  | daf-19 | 38 | 2.11 | 6.1e-04 |
| I$SN\_02 |  | unc-120 (0.55) K02D7.2 C24A1.2 | 81 | 1.43 | 6.2e-04 |
| pTH10730 |  | ceh-36 (-0.52) ceh-53 ceh-45 pha-2 alr-1 dve-1 | 101 | 1.27 | 6.3e-04 |
| MA0046.1 |  | ceh-43 hmbx-1 ceh-53 | 74 | 1.50 | 6.4e-04 |
| pTH2936 |  | nhr-239 | 46 | 1.90 | 6.5e-04 |
| Alx4\_1744 |  | ceh-1 (0.56) lin-39 ceh-45 ceh-10 alr-1 eyg-1 | 44 | 1.94 | 6.7e-04 |
| pTH9163 |  | nhr-3 | 53 | 1.76 | 6.7e-04 |
| MA0498.1 |  | ceh-32 lin-39 | 41 | 2.01 | 6.7e-04 |
| MA0244.1 |  | C48E7.11 | 74 | 1.50 | 6.8e-04 |
| CG31670\_SANGER\_5\_FBgn0031375 |  | CELE\_Y38H8A.5 | 103 | 1.25 | 6.8e-04 |
| Elf3 |  | C24A1.2 | 55 | 1.73 | 6.9e-04 |
| Cdx1\_2245 |  | ceh-13 | 40 | 2.04 | 6.9e-04 |
| Hoxa10\_2318 |  | ceh-24 | 62 | 1.62 | 7.4e-04 |
| Scr\_Cell\_FBgn0003339 |  | cfi-1 php-3 lim-7 lin-39 unc-86 | 82 | 1.42 | 7.5e-04 |
| pTH4325 |  | ceh-18 | 92 | 1.33 | 7.5e-04 |
| Dlx2\_2273 |  | ceh-43 | 42 | 1.98 | 7.5e-04 |
| I$UBX\_01 |  | lin-39 | 96 | 1.30 | 7.8e-04 |
| pTH10717 |  | lsl-1 syd-9 lsy-2 | 74 | 1.49 | 7.9e-04 |
| pTH9957 |  | fkh-9 (0.6) daf-16 irx-1 | 80 | 1.43 | 8.0e-04 |
| V$DELTAEF1\_01 |  | ztf-6 hlh-2 | 42 | 1.97 | 8.1e-04 |
| pTH6447 |  | ceh-19 | 56 | 1.70 | 8.2e-04 |
| Hoxc8\_3429 |  | lin-39 | 53 | 1.75 | 8.3e-04 |
| disco-r-Cl1\_SANGER\_5\_FBgn0042650 |  | lin-31 F55C5.11 | 69 | 1.54 | 8.7e-04 |
| MA0161.1 |  | nfi-1 F49E12.6 | 106 | 1.22 | 9.1e-04 |
| Cdx2\_4272 |  | ceh-13 | 42 | 1.96 | 9.3e-04 |
| V$FREAC3\_01 |  | lin-31 let-381 | 64 | 1.59 | 9.4e-04 |
| pTH5078 |  | ces-2 | 60 | 1.64 | 9.4e-04 |
| MA0118.1 |  | ref-2 | 55 | 1.70 | 9.9e-04 |
| Hoxa7\_3750 |  | lin-39 | 52 | 1.75 | 1.0e-03 |
| TLX1\_f1 |  | ceh-19 | 55 | 1.70 | 1.0e-03 |
| Hoxa7\_2668 |  | lin-39 | 53 | 1.73 | 1.0e-03 |
| pTH9142 |  | ztf-6 C34D1.1 | 97 | 1.29 | 1.0e-03 |
| pTH9245 |  | ceh-18 | 47 | 1.83 | 1.1e-03 |
| BHLHE22\_1 |  | hlh-8 (0.66) lin-31 hlh-15 ngn-1 hlh-32 | 48 | 1.81 | 1.2e-03 |
| En2\_0952 |  | lim-6 lim-7 ceh-16 | 45 | 1.87 | 1.2e-03 |
| Hoxa6\_1040 |  | lin-39 | 56 | 1.68 | 1.2e-03 |
| Hoxa2\_3079 |  | lin-39 | 50 | 1.77 | 1.2e-03 |
| pTH9244 |  | tbx-39 | 69 | 1.52 | 1.2e-03 |
| Etv3 |  | lin-1 | 47 | 1.82 | 1.3e-03 |
| V$PBX1\_02 |  | lin-39 ceh-20 ceh-12 | 53 | 1.72 | 1.3e-03 |
| MA0456.1 |  | ref-2 | 51 | 1.75 | 1.3e-03 |
| MA0035.3 |  | ztf-29 elt-1 | 44 | 1.88 | 1.3e-03 |
| V$GATA3\_01 |  | elt-1 | 37 | 2.06 | 1.3e-03 |
| pTH9289 |  | mab-9 tbx-39 | 56 | 1.67 | 1.4e-03 |
| Hoxd1\_3448 |  | ceh-12 | 40 | 1.97 | 1.4e-03 |
| pTH8679 |  | pax-2 | 41 | 1.95 | 1.4e-03 |
| Nkx1-1\_3856 |  | ceh-30 | 56 | 1.67 | 1.4e-03 |
| Dlx1\_1741 |  | ceh-43 | 42 | 1.92 | 1.4e-03 |
| Mw138 |  | dsc-1 (0.69) ceh-48 | 54 | 1.70 | 1.4e-03 |
| Nkx1-2\_3214 |  | ceh-30 | 99 | 1.26 | 1.5e-03 |
| V$TATA\_C |  | tbp-1 | 105 | 1.21 | 1.5e-03 |
| TBX3\_f1 |  | tbx-39 ceh-45 | 63 | 1.57 | 1.6e-03 |
| Nkx6-1\_2825 |  | cog-1 (0.53) | 83 | 1.38 | 1.6e-03 |
| Mrg1\_2246 |  | ceh-32 ces-1 | 18 | 3.25 | 1.7e-03 |
| scrt\_SOLEXA\_2.5\_2\_FBgn0004880 |  | hlh-8 (0.66) hlh-1 (0.51) ces-1 hlh-15 hlh-2 | 71 | 1.48 | 1.8e-03 |
| CG8765\_SANGER\_5\_FBgn0036900 |  | H20J04.3 | 58 | 1.62 | 1.9e-03 |
| V$LYF1\_01 |  | bed-3 (0.81) nhr-177 F26F4.8 | 25 | 2.54 | 1.9e-03 |
| pTH10041 |  | ztf-29 | 106 | 1.20 | 1.9e-03 |
| pTH3819 |  | ceh-18 | 45 | 1.83 | 2.0e-03 |
| I$DFD\_01 |  | lin-39 | 59 | 1.61 | 2.0e-03 |
| pTH10696 |  | Y44A6D.3 dpy-27 | 33 | 2.14 | 2.0e-03 |
| pTH9480 |  | ces-2 C01B12.2 Y51H4A.4 F23F12.9 | 30 | 2.26 | 2.0e-03 |
| En1\_3123 |  | ceh-16 | 45 | 1.82 | 2.0e-03 |
| Hmbox1\_2674 |  | hmbx-1 | 57 | 1.63 | 2.1e-03 |
| pTH9182 |  | tbx-39 | 56 | 1.64 | 2.1e-03 |
| SH-SY5Y\_GATA2\_UCD |  | elt-1 | 40 | 1.93 | 2.1e-03 |
| pTH6641 |  | lin-31 | 48 | 1.77 | 2.1e-03 |
| pTH10837 |  | T22H9.4 C34H4.5 | 53 | 1.69 | 2.1e-03 |
| pTH10816 |  | dmd-6 | 72 | 1.47 | 2.1e-03 |
| pTH10768 |  | med-2 | 28 | 2.35 | 2.1e-03 |
| pTH5914 |  | attf-1 | 65 | 1.53 | 2.2e-03 |
| pTH6497 |  | lin-31 | 57 | 1.62 | 2.3e-03 |
| Cart1\_1275 |  | lim-7 alr-1 ceh-18 | 42 | 1.87 | 2.4e-03 |
| Elf5 |  | C24A1.2 | 52 | 1.69 | 2.4e-03 |
| Tcf2\_0913 |  | hmbx-1 | 57 | 1.62 | 2.5e-03 |
| MA0083.2 |  | unc-120 (0.55) | 46 | 1.79 | 2.5e-03 |
| Nkx6-3\_3446 |  | cog-1 (0.53) | 56 | 1.63 | 2.5e-03 |
| pTH1014 |  | atf-5 | 28 | 2.32 | 2.6e-03 |
| Hoxb8\_3780 |  | lin-39 | 16 | 3.42 | 2.6e-03 |
| pTH6562 |  | ceh-5 | 61 | 1.57 | 2.6e-03 |
| Jundm2\_0911 |  | fos-1 | 63 | 1.54 | 2.6e-03 |
| Pknox2\_3077 |  | ceh-32 | 17 | 3.25 | 2.7e-03 |
| Hoxc11\_3718 |  | pal-1 ceh-24 | 60 | 1.58 | 2.8e-03 |
| pTH10718 |  | egl-43 | 63 | 1.54 | 2.8e-03 |
| pTH3751 |  | tbx-39 | 56 | 1.62 | 2.8e-03 |
| pTH10779 |  | nhr-182 | 42 | 1.86 | 2.8e-03 |
| MA0451.1 |  | nhr-2 (-0.58) php-3 | 45 | 1.80 | 2.8e-03 |
| MYF6\_1 |  | hlh-1 (0.51) hlh-11 lin-32 | 72 | 1.45 | 2.9e-03 |
| Plagl1\_0972 |  | Y53H1A.2 | 70 | 1.47 | 3.0e-03 |
| Sox4 |  | nhr-100 pop-1 | 66 | 1.51 | 3.1e-03 |
| V$EN1\_01 |  | atf-2 ceh-16 | 40 | 1.89 | 3.1e-03 |
| MA0461.1 |  | hlh-15 hlh-32 | 53 | 1.66 | 3.1e-03 |
| pTH9043 |  | sem-2 | 47 | 1.75 | 3.1e-03 |
| pTH9256 |  | ceh-18 | 91 | 1.30 | 3.2e-03 |
| MA0547.1 |  | skn-1 | 69 | 1.47 | 3.3e-03 |
| MA0544.1 |  | lin-22 gei-11 | 42 | 1.84 | 3.4e-03 |
| Dlx3\_1030 |  | ceh-43 | 73 | 1.44 | 3.4e-03 |
| CG7386\_F10-12\_SANGER\_5\_FBgn0035691 |  | F56D1.1 (-0.55) | 69 | 1.47 | 3.5e-03 |
| pTH9974 |  | hlh-16 ngn-1 hlh-32 | 62 | 1.54 | 3.5e-03 |
| pTH5111 |  | hlh-30 aha-1 | 95 | 1.27 | 3.7e-03 |
| Hoxa5\_3415 |  | lin-39 | 51 | 1.67 | 3.7e-03 |
| pTH3120 |  | K11D2.4 | 97 | 1.25 | 4.1e-03 |
| pTH10645 |  | nhr-100 nhr-7 elt-1 | 47 | 1.73 | 4.1e-03 |
| pTH2846 |  | lin-31 | 45 | 1.76 | 4.3e-03 |
| MA0594.1 |  | lin-39 | 66 | 1.49 | 4.4e-03 |
| GATA5\_1 |  | end-3 elt-1 | 80 | 1.37 | 4.5e-03 |
| Hlxb9\_3422 |  | ceh-12 | 35 | 1.98 | 4.6e-03 |
| pTH5778 |  | egl-5 (0.61) | 55 | 1.60 | 4.7e-03 |
| pTH5812 |  | ceh-14 | 36 | 1.95 | 4.7e-03 |
| K562\_ETS1\_HudsonAlpha |  | slr-2 lin-1 odr-7 nhr-79 nhr-28 | 58 | 1.57 | 4.8e-03 |
| MA0600.1 |  | F52B5.7 daf-19 | 50 | 1.67 | 4.8e-03 |
| Bsx\_3483 |  | ceh-31 | 49 | 1.68 | 4.9e-03 |
| V$GR\_Q6 |  | nhr-255 | 59 | 1.55 | 5.2e-03 |
| pTH9108 |  | nhr-5 (0.52) daf-12 | 104 | 1.20 | 5.2e-03 |
| HLH25 |  | aha-1 lin-22 hlh-27 hlh-28 | 88 | 1.31 | 5.4e-03 |
| V$CDC5\_01 |  | D1081.8 | 88 | 1.31 | 5.4e-03 |
| HSFY2\_1 |  | mab-3 hsf-1 | 65 | 1.49 | 5.4e-03 |
| pTH5083 |  | fos-1 crh-1 | 53 | 1.62 | 5.5e-03 |
| Pou3f1\_3819 |  | ceh-6 | 45 | 1.74 | 5.6e-03 |
| pTH6591 |  | lin-31 | 58 | 1.56 | 5.6e-03 |
| MA0126.1 |  | lin-48 (0.54) dmd-3 pax-3 | 68 | 1.46 | 5.7e-03 |
| pTH9250 |  | dmd-3 C34D1.1 | 69 | 1.45 | 5.8e-03 |
| Sox1\_1 |  | grh-1 (0.69) dmd-4 sox-4 | 59 | 1.54 | 6.0e-03 |
| pTH9247 |  | C34D1.1 | 96 | 1.25 | 6.1e-03 |
| pTH6569 |  | ceh-43 | 49 | 1.67 | 6.2e-03 |
| Caup\_SOLEXA\_FBgn0015919 |  | irx-1 | 53 | 1.61 | 6.2e-03 |
| pTH9246 |  | lin-31 let-381 fkh-10 C34D1.1 | 100 | 1.22 | 6.3e-03 |
| V$GATA1\_05 |  | ceh-32 ceh-34 elt-1 | 63 | 1.50 | 6.3e-03 |
| Hoxd10\_2368 |  | php-3 lin-39 | 35 | 1.94 | 6.4e-03 |
| PO3F1\_f1 |  | ceh-18 tbp-1 | 48 | 1.68 | 6.6e-03 |
| Hoxb5\_3122 |  | lin-39 | 49 | 1.66 | 6.6e-03 |
| pTH10777 |  | dmd-3 | 87 | 1.30 | 6.7e-03 |
| pTH9216 |  | ceh-18 | 50 | 1.64 | 6.9e-03 |
| pTH10772 |  | ceh-52 | 53 | 1.60 | 6.9e-03 |
| pTH1739 |  | npax-1 nhr-255 lin-14 | 59 | 1.53 | 6.9e-03 |
| Mf28 |  | elt-1 | 40 | 1.81 | 7.0e-03 |
| pTH9951 |  | mex-6 | 66 | 1.46 | 7.0e-03 |
| NR2E3\_f1 |  | lin-1 nhr-100 | 55 | 1.58 | 7.1e-03 |
| EPAS1\_si |  | daf-12 ztf-3 ceh-9 hif-1 | 89 | 1.29 | 7.2e-03 |
| pTH9211 |  | C01B12.2 | 37 | 1.87 | 7.3e-03 |
| MA0038.1 |  | lim-6 odd-1 | 50 | 1.64 | 7.4e-03 |
| Atf1\_3026 |  | crh-1 | 63 | 1.49 | 7.4e-03 |
| Osr1\_3033 |  | odd-1 | 90 | 1.28 | 7.5e-03 |
| pTH9260 |  | mel-28 | 83 | 1.33 | 7.5e-03 |
| V$NCX\_01 |  | ceh-19 | 19 | 2.71 | 7.6e-03 |
| pTH10805 |  | ztf-16 | 51 | 1.62 | 7.9e-03 |
| pTH3041 |  | atf-2 | 95 | 1.25 | 8.0e-03 |
| V$YY1\_01 |  | lsy-2 | 48 | 1.66 | 8.2e-03 |
| Irx3\_1 |  | irx-1 | 94 | 1.25 | 8.2e-03 |
| pTH8649 |  | mbr-1 | 49 | 1.64 | 8.3e-03 |
| pTH9314 |  | atf-7 atf-6 fos-1 crh-1 | 54 | 1.58 | 8.3e-03 |
| pTH3046 |  | Y116A8C.22 | 55 | 1.57 | 8.4e-03 |
| Evx1\_3952 |  | ceh-53 | 44 | 1.72 | 8.7e-03 |
| GRHL1\_2 |  | grh-1 (0.69) | 32 | 1.99 | 8.7e-03 |
| CG2052\_SANGER\_2.5\_FBgn0039905 |  | fkh-7 | 43 | 1.73 | 8.7e-03 |
| V$PAX5\_01 |  | pax-2 F45H11.6 | 57 | 1.54 | 8.8e-03 |
| HeLa-S3\_ZNF274\_UCD |  | C28G1.4 (-0.55) | 45 | 1.70 | 9.0e-03 |
| pTH9393 |  | ZC416.1 | 84 | 1.31 | 9.2e-03 |
| YY1\_1 |  | lsy-2 | 100 | 1.21 | 9.2e-03 |
| Vax2\_3500 |  | C02F12.10 | 22 | 2.42 | 9.3e-03 |
| MA0067.1 |  | hlh-30 aha-1 mdl-1 pax-1 | 54 | 1.57 | 9.6e-03 |
| pTH6143 |  | php-3 pal-1 lin-39 ceh-24 | 39 | 1.80 | 9.6e-03 |
| pTH6436 |  | ceh-53 | 44 | 1.71 | 9.7e-03 |
| pTH10623 |  | scrt-1 | 61 | 1.49 | 9.8e-03 |
| EN1\_4 |  | ceh-16 | 75 | 1.37 | 9.9e-03 |
| PTF1A\_f1 |  | lin-32 | 60 | 1.50 | 1.0e-02 |
| HIF1A\_si |  | hif-1 | 87 | 1.29 | 1.0e-02 |
| Mv75 |  | elt-1 | 60 | 1.50 | 1.1e-02 |
| pTH9173 |  | efl-2 | 28 | 2.10 | 1.1e-02 |
| LHX9\_2 |  | ceh-1 (0.56) ceh-14 lin-39 ceh-31 | 55 | 1.55 | 1.1e-02 |
| ZBTB49\_1 |  | aha-1 C46E10.9 | 39 | 1.78 | 1.1e-02 |
| Barx2\_3447 |  | ceh-43 | 59 | 1.50 | 1.2e-02 |
| MA0579.1 |  | D1081.8 | 28 | 2.08 | 1.2e-02 |
| V$AREB6\_01 |  | ztf-6 | 34 | 1.89 | 1.2e-02 |
| CG4854\_SANGER\_10\_FBgn0038766 |  | K11D2.4 | 73 | 1.38 | 1.2e-02 |
| pTH6449 |  | ceh-43 | 56 | 1.53 | 1.2e-02 |
| HMGA1\_f1 |  | Y116A8C.22 | 91 | 1.26 | 1.2e-02 |
| pTH8216 |  | Y116A8C.22 | 99 | 1.21 | 1.2e-02 |
| MITF\_f1 |  | hlh-30 mxl-1 | 56 | 1.53 | 1.2e-02 |
| pTH5916 |  | efl-2 | 45 | 1.67 | 1.2e-02 |
| VDR\_1 |  | cfi-1 nhr-208 | 64 | 1.45 | 1.2e-02 |
| Tcf1\_2666 |  | hmbx-1 | 92 | 1.25 | 1.2e-02 |
| Pou2f1\_3081 |  | ceh-18 | 37 | 1.81 | 1.2e-02 |
| pTH10798 |  | Y75B8A.6 | 95 | 1.23 | 1.3e-02 |
| Hoxa4\_3426 |  | lin-39 | 51 | 1.58 | 1.3e-02 |
| gl\_SANGER\_5\_FBgn0004618 |  | ces-1 che-1 | 58 | 1.50 | 1.3e-02 |
| pTH9149 |  | ztf-30 (0.8) | 79 | 1.33 | 1.3e-02 |
| Hoxd11\_3873 |  | php-3 | 59 | 1.49 | 1.3e-02 |
| rn\_SOLEXA\_5\_FBgn0259172 |  | lin-29 | 81 | 1.32 | 1.3e-02 |
| pTH5781 |  | ceh-32 | 14 | 3.15 | 1.4e-02 |
| V$YY1\_02 |  | lsy-2 | 60 | 1.48 | 1.4e-02 |
| ZBT7A\_f1 |  | klf-1 ZC328.2 | 83 | 1.30 | 1.4e-02 |
| Meox1\_2310 |  | ceh-31 | 52 | 1.56 | 1.4e-02 |
| pTH9353 |  | ceh-51 | 19 | 2.54 | 1.5e-02 |
| tgo\_ss\_SANGER\_5\_FBgn0015014 |  | aha-1 ahr-1 | 49 | 1.60 | 1.5e-02 |
| pTH6482 |  | ceh-19 | 47 | 1.62 | 1.5e-02 |
| PROX1\_1 |  | crh-1 ceh-26 | 93 | 1.24 | 1.5e-02 |
| pTH9220 |  | mbr-1 | 96 | 1.22 | 1.5e-02 |
| LHX2\_f1 |  | ceh-14 | 41 | 1.71 | 1.5e-02 |
| ZN384\_f1 |  | lin-29 K11D2.4 | 65 | 1.43 | 1.6e-02 |
| V$CDPCR3\_01 |  | ceh-48 | 68 | 1.40 | 1.6e-02 |
| MA0146.2 |  | F58G1.2 | 75 | 1.35 | 1.6e-02 |
| pTH10647 |  | nhr-232 | 61 | 1.46 | 1.6e-02 |
| pTH9326 |  | nhr-122 | 37 | 1.79 | 1.6e-02 |
| pTH6106 |  | nhr-182 | 97 | 1.22 | 1.6e-02 |
| Six4\_2860 |  | ceh-32 | 46 | 1.63 | 1.6e-02 |
| pTH10030 |  | xbp-1 | 29 | 1.99 | 1.7e-02 |
| pTH9934 |  | Y53H1A.2 | 45 | 1.64 | 1.7e-02 |
| pTH9907 |  | nhr-34 (0.57) | 44 | 1.65 | 1.7e-02 |
| pTH9097 |  | Y116A8C.22 | 85 | 1.28 | 1.8e-02 |
| V$NKX22\_01 |  | dsc-1 (0.69) ceh-24 | 58 | 1.48 | 1.8e-02 |
| pTH3997 |  | C04F5.9 | 37 | 1.77 | 1.8e-02 |
| Lbx2\_3869 |  | mls-2 | 61 | 1.45 | 1.8e-02 |
| pTH8411 |  | tbx-39 | 37 | 1.77 | 1.8e-02 |
| Srf\_3509 |  | unc-120 (0.55) | 29 | 1.97 | 1.9e-02 |
| MCR\_f1 |  | nhr-255 | 16 | 2.75 | 1.9e-02 |
| Sox17\_2837 |  | sox-4 | 54 | 1.52 | 1.9e-02 |
| Hoxb4\_2627 |  | lin-39 | 11 | 3.64 | 2.0e-02 |
| Gsh2\_3990 |  | ceh-31 | 59 | 1.47 | 2.0e-02 |
| Smad3\_3805 |  | daf-8 | 97 | 1.21 | 2.0e-02 |
| CENPB\_1 |  | F21D5.4 F52B11.1 | 56 | 1.50 | 2.0e-02 |
| pTH9242 |  | mel-28 | 83 | 1.29 | 2.0e-02 |
| pTH6486 |  | nhr-145 | 110 | 1.13 | 2.0e-02 |
| pTH9708 |  | ceh-34 | 50 | 1.56 | 2.1e-02 |
| Vsx1\_1728 |  | alr-1 | 34 | 1.82 | 2.2e-02 |
| V$GATA1\_02 |  | elt-1 | 42 | 1.66 | 2.2e-02 |
| pTH9215 |  | C34D1.1 | 70 | 1.37 | 2.3e-02 |
| pTH9880 |  | end-1 | 49 | 1.56 | 2.3e-02 |
| pTH7032 |  | F52B11.1 | 30 | 1.91 | 2.3e-02 |
| Hoxa3\_2783 |  | lin-39 | 44 | 1.63 | 2.3e-02 |
| Barhl1\_1 |  | ceh-31 | 46 | 1.60 | 2.3e-02 |
| pTH9297 |  | ceh-18 | 87 | 1.26 | 2.4e-02 |
| Pbx1\_3203 |  | ceh-20 | 39 | 1.70 | 2.4e-02 |
| pTH2684 |  | fos-1 | 93 | 1.23 | 2.5e-02 |
| MA0497.1 |  | mef-2 | 84 | 1.28 | 2.5e-02 |
| pTH9254 |  | mel-28 | 58 | 1.46 | 2.5e-02 |
| Tcf7l2\_3461 |  | pop-1 | 55 | 1.49 | 2.5e-02 |
| V$S8\_01 |  | ceh-45 | 38 | 1.72 | 2.5e-02 |
| pTH6508 |  | nhr-36 | 59 | 1.45 | 2.5e-02 |
| Barx1\_2877 |  | ceh-43 | 54 | 1.50 | 2.6e-02 |
| V$VMYB\_01 |  | B0310.2 (0.6) D1081.8 | 53 | 1.51 | 2.6e-02 |
| pTH10027 |  | M03D4.4 | 57 | 1.47 | 2.6e-02 |
| pTH9198 |  | dmd-3 | 44 | 1.62 | 2.6e-02 |
| pTH9188 |  | ceh-32 dmd-5 | 18 | 2.47 | 2.6e-02 |
| pTH10654 |  | ceh-90 | 107 | 1.14 | 2.6e-02 |
| pTH9709 |  | die-1 | 70 | 1.36 | 2.7e-02 |
| FOXC2\_f1 |  | cfi-1 let-381 | 60 | 1.44 | 2.7e-02 |
| pTH6478 |  | lim-7 | 27 | 1.98 | 2.7e-02 |
| V$GATA6\_01 |  | elt-1 | 34 | 1.79 | 2.7e-02 |
| V$AREB6\_02 |  | ztf-6 | 48 | 1.56 | 2.8e-02 |
| MA0027.1 |  | ceh-16 | 50 | 1.53 | 2.8e-02 |
| pTH6268 |  | ceh-2 | 28 | 1.95 | 2.8e-02 |
| MA0032.1 |  | let-381 | 81 | 1.29 | 3.0e-02 |
| Hoxb3\_1720 |  | lin-39 | 45 | 1.59 | 3.1e-02 |
| V$ZID\_01 |  | ztf-28 | 55 | 1.47 | 3.1e-02 |
| pTH1049 |  | elt-1 | 68 | 1.37 | 3.1e-02 |
| pTH9335 |  | mel-28 | 84 | 1.27 | 3.1e-02 |
| Irx2\_0900 |  | irx-1 | 93 | 1.22 | 3.1e-02 |
| pTH10808 |  | ztf-19 | 79 | 1.30 | 3.2e-02 |
| pTH9125 |  | egl-13 K11D2.4 | 98 | 1.19 | 3.3e-02 |
| pTH8985 |  | athp-1 | 95 | 1.21 | 3.3e-02 |
| Elf4 |  | C24A1.2 | 60 | 1.42 | 3.4e-02 |
| Emx2\_3420 |  | ceh-2 | 95 | 1.21 | 3.4e-02 |
| pTH9237 |  | mel-28 | 81 | 1.28 | 3.4e-02 |
| MA0164.1 |  | nhr-100 ztf-11 | 91 | 1.23 | 3.5e-02 |
| pTH5690 |  | ceh-32 | 43 | 1.60 | 3.6e-02 |
| pTH10633 |  | R07H5.10 | 53 | 1.48 | 3.6e-02 |
| pTH8996 |  | sma-4 | 59 | 1.43 | 3.7e-02 |
| pTH9135 |  | pop-1 | 69 | 1.35 | 3.7e-02 |
| pTH9165 |  | ztf-27 (0.63) | 52 | 1.49 | 3.8e-02 |
| Vax1\_3499 |  | C02F12.10 | 60 | 1.42 | 3.8e-02 |
| V$CEBP\_01 |  | C48E7.11 | 42 | 1.60 | 3.9e-02 |
| Hoxc4\_3491 |  | lin-39 | 8 | 4.27 | 4.0e-02 |
| pTH10769 |  | Y48G1C.6 | 62 | 1.39 | 4.2e-02 |
| Spdef\_0905 |  | lin-1 | 91 | 1.22 | 4.2e-02 |
| pTH2933 |  | F58G1.2 | 69 | 1.34 | 4.3e-02 |
| pTH9189 |  | dmd-3 | 40 | 1.62 | 4.3e-02 |
| V$CDPCR1\_01 |  | ceh-48 | 38 | 1.65 | 4.5e-02 |
| Lhx1\_2240 |  | lim-7 | 34 | 1.72 | 4.5e-02 |
| pTH10028 |  | nhr-204 (0.59) | 40 | 1.62 | 4.6e-02 |
| V$IK2\_01 |  | F26F4.8 | 34 | 1.72 | 4.7e-02 |
| pTH9096 |  | T07C12.11 | 80 | 1.27 | 4.7e-02 |
| pTH5257 |  | C48E7.11 | 15 | 2.56 | 4.7e-02 |
| pTH9177 |  | F10B5.3 | 80 | 1.27 | 4.8e-02 |
| Spdef |  | lin-1 | 50 | 1.49 | 4.9e-02 |

### Correlated (and anti-correlated) transcription factors

|  |  |
| --- | --- |
| **Transcription factor** | **Correlation** |
| blmp-1 | 0.91 |
| nhr-23 | 0.90 |
| atf-8 | 0.86 |
| bed-3 | 0.81 |
| lin-26 | 0.80 |
| ztf-30 | 0.80 |
| nhr-31 | 0.72 |
| dsc-1 | 0.69 |
| moe-3 | 0.69 |
| grh-1 | 0.69 |
| nhr-87 | 0.67 |
| lir-1 | 0.67 |
| nhr-25 | 0.67 |
| mls-1 | 0.66 |
| hlh-8 | 0.66 |
| nhr-14 | 0.66 |
| nhr-147 | 0.65 |
| ceh-99 | 0.64 |
| rnt-1 | 0.63 |
| ztf-27 | 0.63 |
| nhr-260 | 0.62 |
| elt-3 | 0.62 |
| ccch-1 | 0.62 |
| nhr-146 | 0.62 |
| madf-1 | 0.62 |
| fkh-2 | -0.55 |
| K11H3.4 | -0.56 |
| lim-4 | -0.56 |
| madf-8 | -0.56 |
| K11D12.12 | -0.57 |
| athp-2 | -0.57 |
| dhhc-10 | -0.57 |
| nhr-2 | -0.58 |
| lin-13 | -0.58 |
| zim-3 | -0.58 |
| isw-1 | -0.59 |
| nhr-215 | -0.59 |
| ceh-91 | -0.59 |
| F17C11.1 | -0.60 |
| ceh-89 | -0.61 |
| K09A11.1 | -0.63 |
| nfya-2 | -0.63 |
| ztf-15 | -0.63 |
| cep-1 | -0.64 |
| T22C8.3 | -0.66 |
| ztf-4 | -0.66 |
| snu-23 | -0.66 |
| hmg-3 | -0.67 |
| aptf-2 | -0.70 |
| wrm-1 | -0.70 |

### ChIP peaks enriched

|  |  |  |  |  |
| --- | --- | --- | --- | --- |
| **Gene** | **Experiment** | **Number of upstream peaks** | **Enrichment** | **FDR corrected p** |
| nhr-23 | NHR-23\_Larvae-L3-stage | 46 | 4.42 | 1.2e-16 |
| nhr-25 | NHR-25\_Larvae-L2-stage | 38 | 4.28 | 7.9e-13 |
| elt-3 | ELT-3\_Embryos | 30 | 4.45 | 3.3e-10 |
| ceh-16 | CEH-16\_Larvae-L2-stage | 21 | 4.98 | 1.1e-07 |
| nhr-28 | NHR-28\_Larvae-L4-stage | 35 | 2.89 | 3.4e-07 |
| fos-1 | FOS-1\_Larvae-L2-stage | 32 | 2.64 | 1.3e-05 |
| sax-3 | SAX-3\_Larvae-L4-stage | 32 | 2.52 | 3.5e-05 |
| sem-4 | SEM-4\_Larvae-L2-stage | 29 | 2.53 | 1.2e-04 |
| ceh-26 | CEH-26\_Late-Embryonic-stage | 26 | 2.66 | 1.8e-04 |
| elt-1 | ELT-1\_Larvae-L2-stage | 10 | 6.52 | 1.9e-04 |
| hpl-2 | HPL-2\_Fed-L1-stage-larvae | 45 | 1.86 | 4.1e-04 |
| fos-1 | FOS-1\_Larvae-L3-stage | 30 | 2.25 | 6.9e-04 |
| dpl-1 | DPL-1\_Larvae-L4-stage | 26 | 2.32 | 1.6e-03 |
| dpl-1 | DPL-1\_Fed-L1-stage-larvae | 18 | 2.91 | 1.9e-03 |
| ham-1 | HAM-1\_Fed-L1-stage-larvae | 37 | 1.81 | 5.2e-03 |
| nhr-21 | NHR-21\_Larvae-L2-stage | 10 | 4.12 | 6.8e-03 |
| ceh-38 | CEH-38\_Larvae-L3-stage | 18 | 2.49 | 1.2e-02 |
| peb-1 | PEB-1\_Larvae-L2-stage | 13 | 2.91 | 1.9e-02 |
| C01B12.2 | C01B12.2\_Larvae-L2-stage | 23 | 2.03 | 2.6e-02 |
| eor-1 | EOR-1\_Larvae-L3-stage | 23 | 2.00 | 3.1e-02 |
| lsy-2 | LSY-2\_Larvae-L1-stage | 25 | 1.89 | 3.9e-02 |
| nhr-6 | NHR-6\_Larvae-L2-stage | 39 | 1.59 | 4.0e-02 |
| R02D3.7 | R02D3.7\_Larvae-L3-stage | 22 | 1.99 | 4.3e-02 |
